# Modern LDL-C estimation equations and therapeutic reclassification: a laboratory medicine perspective

**DOI:** 10.1515/almed-2026-0074

**Published:** 2026-07-06

**Authors:** Jordi Tortosa-Carreres, Begoña Laiz-Marro

**Affiliations:** Laboratory Department, Hospital Universitari i Politècnic La Fe, Valencia, Spain; Clinical Laboratory Medicine Research Group, Health Research Institute Hospital La Fe (IISLaFe), Valencia, Spain

**Keywords:** LDL cholesterol, Friedewald equation, Martin-Hopkins, Sampson equation, cardiovascular risk, therapeutic reclassification

To the Editor,

Recent European recommendations for dyslipidemia management have consolidated progressively intensive low-density lipoprotein (LDL-C) targets, including levels below 55 mg/dL in very high-risk patients and below 40 mg/dL in extreme-risk settings defined by recurrent cardiovascular events [1], 2]. The widespread use of high-intensity statins, ezetimibe, and PCSK9 inhibitors has progressively shifted clinical decision-making toward very low LDL-C concentrations, increasing the clinical relevance of small differences in LDL-C estimation [2].

Much of the evidence supporting current therapeutic thresholds was generated in an analytical context dominated by the Friedewald equation [3]. Introduced in 1972 as an alternative to ultracentrifugation [4], Friedewald estimation became the standard approach in routine practice and most randomized clinical trials, including landmark studies such as 4S and FOURIER [3]. Although current LDL-C targets were largely informed by outcome trials conducted in an analytical environment where Friedewald estimation predominated, the assumptions underlying this equation differ from current practice, characterized by lower LDL-C targets, frequent hypertriglyceridemia, and widespread use of non-fasting samples [1], 2]. Under these conditions, Friedewald estimation tends to underestimate LDL-C at low concentrations, precisely where contemporary therapeutic decisions are concentrated [5], 6].

To overcome these limitations, several alternative equations have been proposed. Both incorporate triglycerides and non-HDL cholesterol, albeit through different approaches: the Martin–Hopkins equation replaces the fixed TG/5 ratio with an adjustable factor derived across a continuum of lipid values [6], whereas the Sampson equation applies a multivariable model to better estimate the contribution of triglyceride-rich lipoproteins [7]. The analytical performance of these newer equations has been extensively evaluated, consistently demonstrating improved agreement with β-quantification, particularly in patients with hypertriglyceridemia or low LDL-C concentrations [5], 6], 8].

Recent ACC/AHA guideline updates increasingly favor the use of alternative LDL-C estimation methods over the Friedewald equation in routine clinical practice [2]. Similarly, Spanish consensus recommendations also support the use of alternative equations in selected settings and reinforce the incorporation of non-HDL cholesterol into lipid interpretation [9].

Nevertheless, this methodological transition raises an issue beyond analytical refinement, and one that has received comparatively less attention. Replacing the Friedewald equation with more accurate methods may systematically alter therapeutic classification without necessarily reflecting true changes in atherogenic burden. In other words, we may be redefining who is “at target” without redefining the target itself.

Previous evidence has begun to characterize this phenomenon from complementary but distinct perspectives. Martin et al., in over 1.3 million lipid profiles classified into treatment strata defined by 30 mg/dL increments (<70, 70–99, 100–129 mg/dL and above), reported that 14.6 % of patients were reclassified into a different stratum when Friedewald estimation was replaced by direct ultracentrifugation measurement, predominantly upward (11.3 %) [3]. Samuel et al., in a database of over five million patients, found that 19.8 % of individuals with Friedewald LDL-C <70 mg/dL were reclassified above this threshold by the Martin–Hopkins equation, rising to nearly half among patients with triglyceride levels of 150–399 mg/dL; the Sampson equation produced a directionally consistent but more modest effect (13.9 %) [8]. Cartier et al. moved closer to clinical practice, evaluating the Martin–Hopkins equation in 4,150 high-risk patients defined by diabetes (HbA1c ≥6.5 %) and/or established cardiovascular disease with triglycerides <400 mg/dL. They reported LDL-C values on average 7.3 % higher with Martin–Hopkins than Friedewald, with similar target attainment between equations at normal triglyceride levels, but with Martin–Hopkins showing better concordance with Non-HDL-C and ApoB as triglyceride levels increased [10].

**Figure 1:**
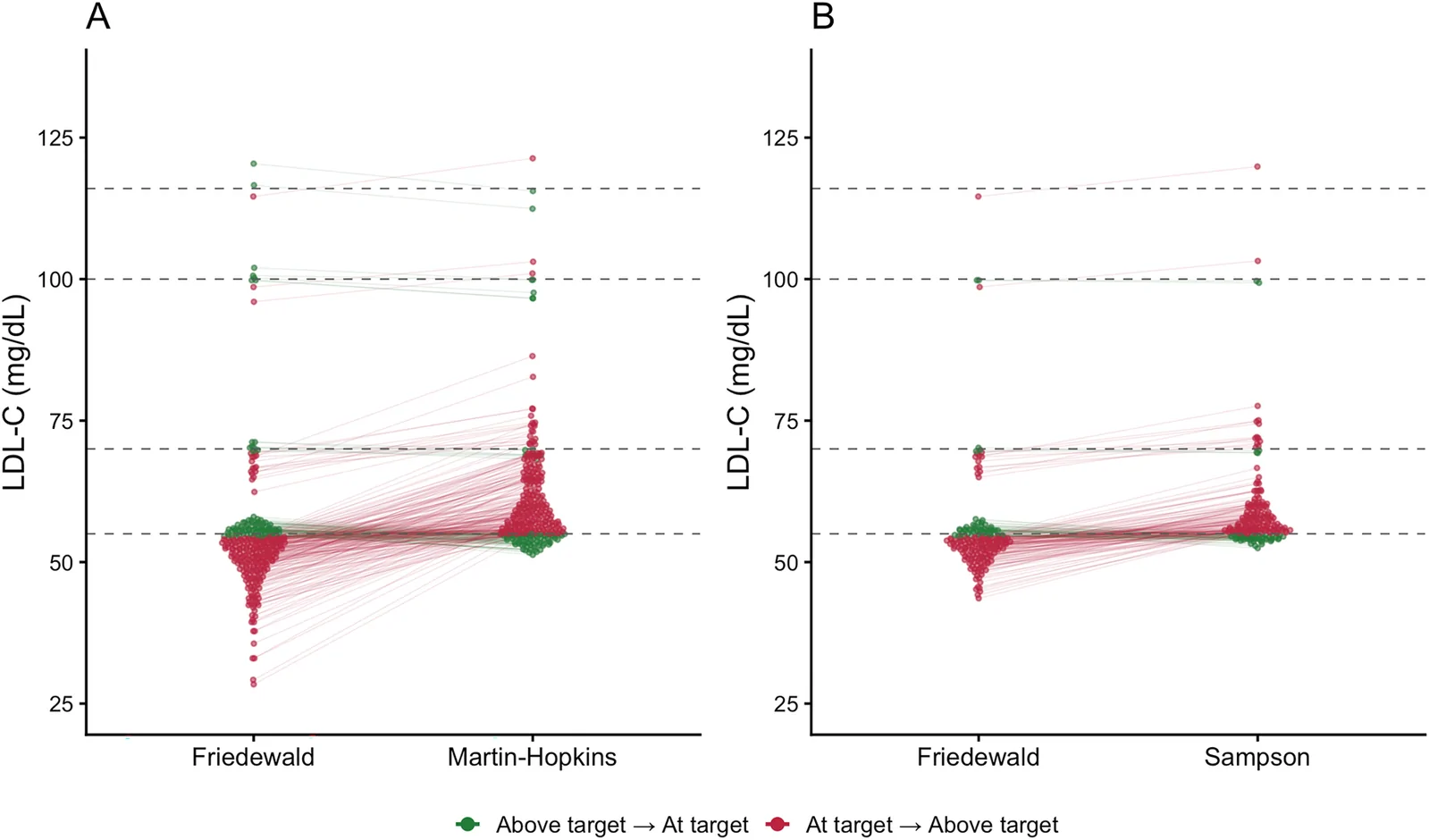
Therapeutic reclassification after LDL-C recalculation using alternative estimation equations. (A) Friedewald vs. Martin–Hopkins equation. (B) Friedewald vs. Sampson equation. Only patients undergoing therapeutic reclassification after LDL-C recalculation are represented. Dashed horizontal lines indicate contemporary LDL-C therapeutic thresholds used in cardiovascular risk stratification (55, 70, 100, and 116 mg/dL).

In our retrospective cohort of 9,405 primary care patients with triglyceride levels <400 mg/dL, enriched with high- and very-high cardiovascular risk individuals (20.1 and 77.1 %, respectively), cardiovascular risk categories and LDL-C therapeutic targets were assigned according to ESC/EAS 2025 recommendations using a laboratory-based CDSS integrating SCORE2/SCORE2-OP/SCORE2-Diabetes models adjusted for lipoprotein(a) together with direct clinical high-risk conditions [1]. Recalculation using alternative equations produced markedly asymmetric shifts: using the Martin–Hopkins equation, 18.3 % of patients initially classified as at target by Friedewald were reclassified as above target, whereas only 0.95 % of those initially above target were reclassified as controlled. The Sampson equation produced directionally consistent but less pronounced findings (11.4 and 0.57 %, respectively). As illustrated in Figure 1, most reclassified patients clustered around contemporary therapeutic thresholds. This asymmetric pattern indicates that equation replacement predominantly generates upward therapeutic reclassification rather than bidirectional redistribution: patients near a therapeutic cut-off may cross classification boundaries after recalculation, whereas those with clearly uncontrolled LDL-C remain above target regardless of the estimation method applied.

Our results offer further evidence on the clinical impact of LDL-C equation selection in a real-world primary care cohort formally stratified according to current ESC/EAS recommendations, extending previous analyses – limited to historical treatment strata up to 70 mg/dL – to the 55 mg/dL threshold, where the known tendency of Friedewald to underestimate LDL-C is most pronounced and, therefore, equation-related discrepancies are greatest [5], 6].

The predominance of high- and very-high cardiovascular risk individuals could be viewed as a limitation in terms of generalizability. However, as outlined above, this represents precisely the population in which equation-related reclassification carries the greatest clinical consequence and where focused evaluation is most warranted, likely maximizing the detection of clinically relevant reclassification events in the stratum where therapeutic decisions are most critical. We lacked ApoB measurements to formally assess whether equation-driven reclassification reflected improved atherogenic concordance, which represents a limitation of the present analysis. Further studies should evaluate whether similar effects are observed in populations with greater representation of low- and moderate-risk individuals, who, albeit a minority in our cohort (63 and 210 patients, respectively), constitute a considerable absolute number for exploratory analyses.

Beyond reclassification, the progressive adoption of alternative equations introduces another fundamental concern: a potential disconnect between historical therapeutic cut-offs and contemporary LDL-C estimation strategies, given that current LDL-C targets were largely informed by outcome trials conducted in an analytical environment where Friedewald estimation predominated. In this scenario, the equation used is no longer a mere analytical detail but a variable that operationally defines therapeutic control itself.

The transition toward more accurate LDL-C estimation methods represents a paradigmatic shift that requires alignment between contemporary estimation strategies and the therapeutic thresholds historically framed within a Friedewald-based analytical context. Whether the progressive adoption of alternative equations will ultimately necessitate a formal re-evaluation of established therapeutic cut-offs remains an open question that will likely be informed by accumulating real-world evidence. Importantly, these considerations should not be interpreted as a barrier to the implementation of newer equations, whose superior analytical performance has been consistently demonstrated. Rather, they highlight the need for a structured transition process, including transparent reporting of the equation used, local assessment of its impact on therapeutic classification, and careful consideration of implications for longitudinal patient monitoring and treatment intensification.

Non-HDL cholesterol may serve as a complementary longitudinal reference when LDL-C values and therapeutic classification are modified by the estimation method rather than by true biological variation, since it is independent of any specific estimation equation. At the reporting level, predefined interpretative comments including the equation used and a warning of potential method-related reclassification represent a feasible first step. When cardiovascular risk stratification is available – as in laboratory settings integrated with a Clinical Decision Support System – personalized reports incorporating therapeutic targets, the estimation method used, and longitudinal interpretation may further facilitate a clinically meaningful transition toward newer LDL-C estimation methods.
